# Supplementary material for: Patterns of Intron Gain and Loss in Fungi
Source: PLoS Biol. 2004 Nov 30;2(12):e422. doi: 10.1371/journal.pbio.0020422 (PMC532390; doi:10.1371/journal.pbio.0020422)
Supplement: Table S1 — Also available at http://genes.mit.edu/NielsenEtAl/. (4.3 MB ZIP). [file pbio.0020422.st001.zip › NielsenEtAl/html/1285.html]

AN0871.1.NCU07259.1.MG10490.1.FG09635.1


```
 CLUSTAL W (1.82) Multiple Sequence Alignments - Introns Inserted


Sequence 1: NCU07259.1	401 aa
Sequence 2: MG10490.1	394 aa
Sequence 3: FG09635.1	391 aa
Sequence 4: AN0871.1	397 aa
Alignment Length: 404 aa
Number Identitical Residues: 233 aa
Alignment Score (without introns) 10309


MG10490.1 	MQLSRLLQLPTLLAAALLAVAPPVAAEHTSNWAVLVCTSRFWFNYRHLANVLSMYRTVKR
NCU07259.1	MKLSNILRLPALLGAMLLGTAAVVEAEHTSNWAVLVCTSRFWFNYRHLANVLSIYRTVKR
FG09635.1 	MKLSN-LWFHALVAAALFATT--AFAEHTSNWAVLVCTSRFWFNYRHLANVLSMYRTVKR
AN0871.1  	--MSPYFGLQALPFLLSLLFAVFASAEHTSNWAVLVATSRFWFNYRHLANVLSLYRTVKR
          	  :*  : : :*     :  :  . ***********.****************:******

MG10490.1 	LGIPDSQIILMLPDDMACNPRNAFPGTVYSNAERAVDLYGDNIEVDYRGYEVTVENFIRL
NCU07259.1	LGIPDSQIILMLPDDMACNPRNAFPGTVYSNADRAVDLYGDNIEVDYRGYEVTVENFIRL
FG09635.1 	LGIPDSQIILMLPDDMACNPRNAFPGTVYSNSDRAVDLYGDNIEVDYRGYEVTVENFIRL
AN0871.1  	LGIPDSQIILMLPDDMACNPRNAFPGTVYSNADRAVDLYGDNIEVDYRGYEVTVENFIRL
          	*******************************::***************************

MG10490.1 	LTDRVGDEMPRSKRLLTDDRSNILVYMTGHGGNEFLKFQDAEEIGAFDLADAFEQMWEKK
NCU07259.1	LTDRVGDEMPRSKRLLTDDRSNILVYMTGHGGNEFLKFQDAEEIGAFDLADAFEQMWEKK
FG09635.1 	LTDRVGAEMPRSKRLLTDDRSNILVYMTGHGGNEFLKFQDAEEIGAFDLANAFEEMWEKK
AN0871.1  	LTDRLDEDVPRSKRLGSDAGSNVLVYMTGHGGDQFLKFQDAEEIGAWDLADAFGQMWEKK
          	****:. ::****** :*  **:*********::************:***:** :*****

MG10490.1 	R2YNEILFMIDTCQANTMLTKLYSPNIIATGSSELDQSSYSHHADNDVGVAVIDRYTYYN
NCU07259.1	R2YHEILFMIDTCQANTMYSKLYSPNIIATGSSELDQSSYSHHADNDIGVAVIDRYTYYN
FG09635.1 	R2YHEILFMIDTCQANTMYSRLYSPNIIATGSSKLDQSSYSHHADNDVGVAVIDRYTYYN
AN0871.1  	R~YHELLFMIDTCQANTMYTHFYSPNIIATGSSELDQSSYSHHADNDVGVAVIDRWTYYV
          	* *:*:************ :::***********:*************:*******:*** 

MG10490.1 	LDFLENEVKDINSKKTIGDLFDSYTFEKIHSHAGMRYDLFPGGADAARAKLLTDFFGNVQ
NCU07259.1	LEFLENEVKDTSSKKTVGDLFDSYTYEKIHSNAGVRYDLFPGGAEAARSRLVTDFFGNVQ
FG09635.1 	LEFLENNVKDLNSQKTVGELFDSYDYSKIHSHAGVRYDLFPGGEENARSRLITDFFGNIQ
AN0871.1  	LEFLENRVTSLDSKQTLGDLFDSYDETKIHSNPGVRWDLFPGGEQEGRLRTVVDFFGNVQ
          	*:****.*.. .*::*:*:*****   ****:.*:*:****** : .* : :.*****:*

MG10490.1 	NVEVDANVT---TGLDEDLIALSKKIAALKAKAESEESAAASENRESS--TTAAPKDNQS
NCU07259.1	NVEVDGSKN---TTLEEDLLALSKTIAALREKAGEEEAISVGKGNAARNETKKETKNETL
FG09635.1 	NVEVDRSGN---LTLEEDLLALSKKIAVLQQKEAEASKKEAGEKVSAQAPASVPTGS---
AN0871.1  	SVEIEKADADEPGSLKEDLIEIAQLVEKWRTFSQDYAVANSTSQQEETRSAPAYEVK---
          	.**::    ...  *.***: ::: :   :    .       .       :     .   

MG10490.1 	LPKRSQITKPLTDDNYWTKKIISATAISGCALLWGLSSYL-----
NCU07259.1	LTHKSQFAKPLTDDDWLVKKLSALGAVVGCAVLWQMGSWLESAQK
FG09635.1 	-VRKIQKPKALTDDNWWTKKVVGATALAGCGLLWALGSFLEG---
AN0871.1  	--KRNVGPARLTDETPWEKRLIGLSILGACAAVWFTGSTLGKTMA
          	  ::   .  ***:    *:: .   : .*. :*  .* *  :
```
